# Prospective association between standing balance and cognitive function in middle-aged and older Chinese adults

**DOI:** 10.3389/fpsyg.2022.931216

**Published:** 2022-09-26

**Authors:** Jingzheng Yan, Fangyun Luan, Meijuan Wang, Wenshuo Dong, Xinyue Zhang, Mengli Li, Yingjuan Cao

**Affiliations:** ^1^School of Nursing and Rehabilitation, Cheeloo College of Medicine, Shandong University, Jinan, China; ^2^Department of Emergency, Qilu Hospital, Shandong University, Jinan, China; ^3^Department of Nursing, Qilu Hospital, Shandong University, Jinan, China

**Keywords:** aging, cognitive function, balance, prospective study, China

## Abstract

**Objective:**

To investigate the association of standing balance with cognitive functions and the rate of cognitive decline among middle-aged and older Chinese adults.

**Methods:**

Participants were selected from China’s Health and Retirement Longitudinal Study. A total of 8,499 subjects aged ≥45 years who participated in wave 1 to wave 3 surveys were included in the final analysis. Standing balance was measured using the tandem test, and participants were categorized into two groups according to their ability to maintain standing balance. Cognitive functions were assessed in three domains: episodic memory, mental status, and global cognition. The associations between standing balance scores, cognitive scores, and the rate of cognitive decline were evaluated using linear regression and linear mixed models.

**Results:**

Compared with participants who successfully completed the standing balance test, those who were unable to complete the test had lower scores on episodic memory [β = −0.18; 95% confidence interval (CI): −0.24, −0.11], mental status (β = −0.28; 95% CI: −0.37, −0.19), and global cognition (β = −0.51; 95% CI: −0.65, −0.38) after 4 years of follow-up. In addition, the rate of decline in mental status and global cognition increased by 0.10 (β = 0.10; 95% CI: 0.07, 0.13) and 0.08 (β = 0.08; 95% CI: 0.04, 0.12) units, respectively, in participants who were unable to complete the test compared with their counterparts.

**Conclusion:**

Good standing balance was significantly associated with higher cognitive function and a lower decline in mental status and global cognition in middle-aged and older Chinese adults.

## Introduction

Cognitive impairment, which was an intermediate stage between normal aging and dementia, is an important health problem in China (Petersen et al., 1999, 2001, 2014; Xue et al., 2018). The prevalence of cognitive impairment was reported to be 14.7% in the old Chinese (Xue et al., 2018). Considering the large aging population in China, many people might suffer from cognitive impairment. Cognitive impairment could increase the risk of dementia, disability, and mortality, which highlight the importance of identifying potential risk factors for cognitive impairment (Shimada et al., 2016; van Rossum and Koek, 2016; Bae et al., 2018).

Physical function was reported to be closely related to cognitive impairment (Tangen et al., 2014). The physiological connection between physical function and cognitive impairment might be that physical function depends on the signals and feedback from the vestibular system and brain (Massion, 1998; van der Kooij et al., 1999; Cullen, 2012). Previous studies found that the occurrence of physical function impairment precedes the occurrence of cognitive impairment, which suggested that measurement of physical function might be a useful tool in screening future cognitive impairment (Camicioli et al., 1998; Buracchio et al., 2010).

Standing balance is an important component of physical function. Several studies have examined the associations between standing balance and cognitive performance (Leandri et al., 2009; Wilkins et al., 2013; Bolandzadeh et al., 2015; Bahureksa et al., 2017; Goto et al., 2018). The Cardiovascular Health Study found a positive correlation between standing balance and cognition in older adults (Meunier et al., 2021). However, this study was conducted in elderly patients with cardiovascular diseases, and the findings were not validated in a large cohort of middle-aged and old adults. In addition, it is also unclear whether this association also existed in the middle-aged and old Chinese, and whether the decline rate of cognition differed in people with different ability of standing balance.

This prospective study examined the association between standing balance and cognitive function in a large cohort of middle-aged and older Chinese adults from the Health and Retirement Longitudinal Study (CHARLS). The CHARLS was chosen because the cohort underwent several cognitive tests (Zhao et al., 2014).

## Materials and methods

### Study population

The analyzed data were obtained from the CHARLS and are publicly available. The CHARLS is a national longitudinal survey conducted by the National School of Development at Peking University involving a large cohort of individuals aged ≥45 years in China. The survey had a multi-wave design and included several cognitive tests. Researchers can access the data by providing basic information online and signing a data use agreement. The survey protocol and implementation were described in a previous study (Zhao et al., 2014).

**FIGURE 1:**
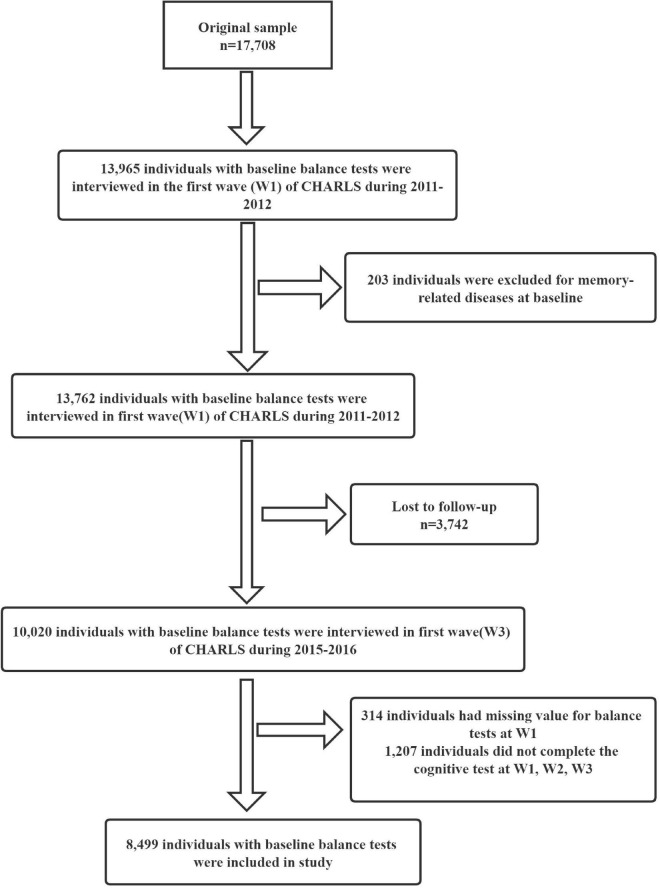
Flowchart of patient selection.

Figure 1 shows the flowchart of patients selection. The 2011 baseline survey (wave 1) recruited 17,708 participants from 28 Chinese provinces, 150 counties/districts, and 450 areas, and the overall response rate was 80.5%. In this cohort, 13,965 (78.9%) participants underwent physical tests, and 203 participants were excluded because of memory-related diseases, resulting in a sample size of 13,762 participants at baseline. Follow-up interviews were conducted in 2013 (wave 2) and 2015 (wave 3). Among the enrollees, 3,742 were lost to follow-up, and 10,020 were re-interviewed in wave 3. Of these, 314 had missing data on the balance test in the wave 1 survey, and 1,207 did not undergo the cognitive test. Thus, 8,499 people were included in the analysis.

This research was approved by the Research Ethics Committee of Peking University. All participants gave written informed consent. The quality of observational research was assessed using Strengthening the Reporting of Observational Studies in Epidemiology guidelines.

### Cognitive function assessment

Cognitive function at baseline and follow-up was measured using face to face questionnaire test and involved in three domains: mental status, episodic memory, and visuospatial working memory. Mental status was assessed using the Cognitive Status test (Meng et al., 2019). Participants were asked to do a serial subtraction from 100 by 7 (up to five times) and date recall. The scores of TICS-10 ranges from 0 to 10 (Folstein et al., 1975). Episodic memory and visuospatial working memory were evaluated using a modified questionnaire based on American Health and Retirement Study. In assessing episodic memory, 10 unrelated Chinese words were read to each participant, and then each participant was asked to recall these words immediately (immediate word recall) and 4 min later (delayed word recall) as much as possible. The ability of episodic memory was evaluated based on the average number of words recalled correctly by participants in immediate recall and delayed recall (Lei et al., 2012). The score of episodic memory ranged from 0 to 10 (McArdle et al., 2007). Visuospatial working memory was assessed by asking participants to redraw a picture of overlapping pentagons on the paper, and participants who were able and unable to redraw were given a score of 1 and 0, respectively (Mathuranath et al., 2000). The total score of cognitive functioning (global cognition) was calculated as the sum of the scores of mental status, episodic memory, and visuospatial working memory, ranging from 0 to 21. Higher scores indicated better cognitive function.

### Balance tests

The static observation method was used in our study to assess the ability of balance. Participants were asked to maintain three standing positions: side-by-side, semi-tandem (heel and toe side-by-side), and full-tandem (heel in front of the toe). Participants were first asked to stand in a semi-tandem position for 10 s. Those who successfully completed this task were then asked to maintain a full-tandem position for 30 s (participants aged ≥70 years) or 60 s (participants aged <70 years). The participants who were unable to perform these two tests were asked to stand with their feet side-by-side for 10 s. Given the small number of participants who successfully completed the latter test, the participants was divided into two groups: participants who could maintain a full-tandem position and those who could not.

### Covariates

Covariates included sociodemographic characteristics (age, sex, county of residence, marital status, and education level), lifestyle variables [sleep duration, smoking status, alcohol consumption, engagement in social activities, and activities of daily living (ADLs)], and clinical characteristics [depression, body mass index (BMI, in kg/m^2^), and the number of chronic diseases]. Chronic diseases included hypertension, dyslipidemia, diabetes, heart disease, stroke, chronic lung disease, asthma, liver disease, cancer, digestive disease, kidney disease, arthritis, psychiatric disease, and memory-related disease. Marital status was categorized as married, single, divorced, widowed, or never married. Subjects were categorized into four groups according to their educational level: illiterate, primary school, middle school, and high school or above. Depression symptoms were assessed using the short depression scale of the Center for Epidemiologic Studies (Andresen et al., 1994). Social behaviors included socializing with friends, playing cards or mahjong with other people, visiting a local community club, participating in community programs, volunteering or doing charitable work, caring for sick or disabled adults, attending educational or training courses, and surfing the web. ADLs included eating, dressing, walking, and personal hygiene. ADL was scored as 1 if any of these tasks was impaired and 0 if none was impaired.

### Statistical analyses

The participants were categorized according to their ability to complete the balance tests. Continuous and categorical variables were presented as means ± standard deviations and frequency (percentages), respectively. The association between balance test scores and cognitive function scores at baseline (wave 1 survey) was evaluated using linear regression. The relationship between standing balance scores and cognitive function scores at baseline was adjusted for age, sex, marital status, education, county of residence, alcohol consumption, smoking status, sleep duration, ADLs, engagement in social activities, BMI, depression, chronic diseases, and other variables (years since baseline) and assessed using linear mixed models. To examine the association between baseline test scores and the rate of cognitive decline, we added an interaction term—years since baseline—to the linear mixed models and adjusted for potential confounders. Statistical analyses were performed using R version 4.0.2 (R Foundation) and STATA version 13.0 (StataCorp). Two-sided *p*-values of less than 0.05 were considered statistically significant.

## Results

### Baseline characteristics

A total of 8,499 participants from the wave 1 survey were included in the analysis, and 2,099 (23.6%) participants failed to complete the tandem test. The baseline characteristics of participants are shown in Table 1. The participants unable to complete the tests were older, less educated, less likely to participate in social activities, more likely to be female, more likely to smoke and consume alcohol, and more likely to have depressive symptoms and multiple chronic diseases. Moreover, this group had less total sleep time, more limitations in ADLs, and lower scores on episodic memory, mental status, and global cognition.

**TABLE 1:** Baseline characteristics of the study participants.

Characteristic	Unable to maintain standing balance (*n* = 2,009)	Able to maintain standing balance (*n* = 6,490)	*P*-value
Age, years	66.7 ± 9.2	63.8 ± 8.7	<0.001
Sex, *n* (%)			<0.001
Female	1,347 (67.0)	3,275 (50.5)	
Male	662 (33.0)	3,215 (49.5)	
County of residence, *n* (%)			0.972
Urban	132 (6.6)	430 (6.6)	
Rural	1,877 (93.4)	6,060 (93.4)	
Educational level, *n* (%)			<0.001
Illiterate	759 (37.8)	1,541 (23.7)	<0.001
Primary school	808 (40.2)	2,846 (43.9)	
Middle school	328 (16.3)	1,411 (21.7)	
High school or higher	114 (5.7)	692 (10.7)	
Marital status, *n* (%)			<0.001
Married	1,637 (81.5)	5,589 (86.1)	
Single, widow, divorced, and never married	372 (18.5)	901 (13.9)	
Smoking status *n*, (%)			<0.001
Current smoker	505 (25.1)	2,063 (31.8)	
Never smoker	1,354 (67.4)	3,859 (59.5)	
Ever smoker	150 (7.5)	568 (8.8)	
Alcohol consumption, *n* (%)			<0.001
Never	390 (19.4)	1,715 (26.4)	
Less than once a month	118 (5.9)	546 (8.4)	
At least once a month	1,501 (74.7)	4,229 (65.2)	
Body mass index (kg/m^2^)	23.8 ± 4.2	23.6 ± 3.9	0.018
Sleep duration (hours)	6.1 ± 1.9	6.5 ± 1.8	<0.001
Engagement in social activities	0.6 ± 0.8	0.8 ± 0.9	<0.001
Limitations in activities of daily living, *n* (%)			<0.001
No	1,532 (76.3)	5,754 (88.7)	
Yes	477 (23.7)	736 (11.3)	
Number of chronic diseases, *n* (%)			<0.001
0	520 (25.9)	2,263 (34.9)	
1	574 (28.6)	1,999 (30.8)	
2	424 (21.1)	1,231 (19.0)	
≥3	491 (24.4)	997 (15.4)	
CESD-10	10.9 ± 5.1	9.6 ± 4.7	<0.001
Cognition			
Global cognition	9.6 ± 4.3	11.3 ± 3.9	<0.001
Episodic memory	3.0 ± 1.8	3.4 ± 1.8	<0.001
Visuo-spatial working memory	0.5 ± 0.5	0.7 ± 0.5	<0.001
Mental status	6.1 ± 2.9	7.2 ± 2.7	<0.001

Continuous and categorical variables were presented as means ± standard deviations and frequency (percentages), respectively. CESD-10, Center for Epidemiologic Studies Depression Scale.

### Association between standing balance score and cognitive scores at baseline

The association between balance test scores and cognitive function scores is shown in Table 2. Compared with participants who successfully completed the balance test, those who were not able to complete the test had lower scores on episodic memory (β = −0.17; 95% CI: −0.25, −0.08), mental status (β = −0.41; 95% CI: −5.26, −0.30), and global cognition (β = −0.62; 95% CI: −0.79, −0.46). These associations remained significant even after adjusting for age, education, marital status, alcohol consumption, smoking status, county of residence, sleep duration, ADLs, participation in social activities, depression, BMI, and the number of chronic diseases.

**TABLE 2:** Cross-sectional association between standing balance scores and cognitive function scores at baseline (wave 1).

Cognitive function	Model 1	Model 2	Model 3
			
	β (95% CI)	β (95% CI)	β (95% CI)
Episodic memory	−0.46 (−0.55, −0.37)*	−0.23 (−0.31, −0.14)*	−0.17 (−0.25, −0.08)*
Mental status	−1.11 (−1.25, −0.98)	−0.50 (−0.62, −0.39)*	−0.41 (−5.26, −0.30)*
Global cognition	−1.72 (−1.92, −1.52)*	−0.78 (−0.95, −0.61)*	−0.62 (−0.79, −0.46)*

*p < 0.05, β, beta coefficient; CI, confidence interval. Model 1 was not adjusted for potential confounders. Model 2 was adjusted for age, education, marital status, alcohol consumption, smoking status, and county of residence. Model 3 was adjusted for model 2 covariates plus sleep duration, limitations in activities of daily living, participation in social activities, depression, body mass index, and number of chronic diseases.

### Association between standing balance scores and cognitive scores at follow-up

The relationship between balance test scores and cognitive function scores at follow-up is shown in Table 3. Compared with participants who successfully complete the test, those who were unable to complete the test had lower scores in episodic memory (β = −0.18; 95% CI: −0.24, −0.11), mental status (β = −0.28; 95% CI: −0.37, −0.19), and global cognition (β = −0.50; 95% CI: −0.64, −0.37) after 4 years of follow-up.

**TABLE 3:** Associations between standing balance scores and cognitive function scores from baseline (wave 1) to follow-up (wave 3).

Cognitive function	Model 1	Model 2	Model 3
			
	β (95% CI)	β (95% CI)	β (95% CI)
Episodic memory	−0.48 (−0.56, −0.41)*	−0.20 (−0.26, −0.14)*	−0.18 (−0.24, −0.11)*
Mental status	−0.95 (−1.06, −0.83)*	−0.33 (−0.42, −0.24)*	−0.28 (−0.37, −0.19)*
Global cognition	−1.59 (−1.77, −1.41)*	−0.59 (−0.73, −0.45)*	−0.51 (−0.65, −0.38)*

*p < 0.05, β, beta coefficient; CI, confidence interval. Model 1 was not adjusted for confounders. Model 2 was adjusted for age, education, marital status, alcohol consumption, smoking status, and county of residence. Model 3 was adjusted for model 2 covariates plus sleep duration, limitations in activities of daily living, participation in social activities, depression, body mass index, and number of chronic diseases.

### Association between standing balance scores and rates of cognitive decline

Significant interactions between balance test scores and years since baseline were observed in cognitive function (Table 4). Compared with participants who successfully complete the balance tests at baseline, those who were unable to completed the tests had rates of cognitive decline in increase by 0.10 (β = 0.10; 95% CI: 0.07, 0.13) units in mental status and 0.08 (β = 0.08; 95% CI: 0.04, 0.12) units in global cognition per year.

**TABLE 4:** Changes in cognitive functions according to standing balance scores.

Cognitive function	Intercept	Time since baseline	Balance test scores × time since baseline
			
	β (95% CI)	β (95% CI)	β (95% CI)
Episodic memory	−4.75 (−5.02, −4.47)	0.05 (0.03, 0.07)	−0.02 (−0.04, 0.01)
Mental status	−5.64 (−6.04, −5.22)	0.34 (0.31, 0.36)	0.10 (0.07, 0.13)*
Global cognition	−10.98 (−11.59, −10.38)	0.40 (0.36, 0.44)	0.08 (0.04, 0.12)*

*p < 0.05, β, beta coefficient; CI, confidence interval. Adjusted for age, education, marital status, alcohol consumption, smoking status, county of residence, sleep duration, limitations in activities of daily living, participation in social activities, time since baseline, depression, body mass index, and number of chronic diseases.

## Discussion

This study evaluated the longitudinal association between standing balance and cognitive function. The results showed that poor standing balance was associated with lower cognitive function, including episodic memory, mental status, and global cognition. In addition, poor balance was associated with a faster decline in mental function and global cognition.

These results demonstrate that poor balance is associated with lower cognitive performance in the Chinese population, consistent with other studies (Goto et al., 2018; Meunier et al., 2021). For instance, a population-based longitudinal study assessed the association between physical performance (4 m walk, five-chair stand, handgrip, and standing balance) and dementia and found that balance was strongly associated with new-onset dementia (hazard ratio = 1.9–2.5, *p* = 0.02) (Bullain et al., 2016). In addition, the Cardiovascular Health Study involving 4,811 participants aged ≥67 years and followed for 6 years found that individuals with poor balance had a faster rate of cognitive decline after adjusting for confounders (β = −0.21, 95% CI = −0.37, −0.05) compared with the control group (Meunier et al., 2021). Our results demonstrated that balance test scores were an independent predictor of changes in cognitive performance in middle-aged and older adults in China. Our findings suggested that interventions in preventing cognitive impairment should be adopted at the same time if participants had limitation in physical function.

The mechanisms underlying the association between standing balance and cognitive decline remain unclear. Static balance depends on the cooperation between sensory (vestibular, proprioceptive, and visual), musculoskeletal, nervous, and cardiovascular systems (Hausdorff et al., 2003; Sturnieks et al., 2008; Abate et al., 2009; Wu et al., 2021). Impairments in brain structures and functions may explain the connection between cognitive function and static balance (Kido et al., 2010; Makizako et al., 2013). In addition, this association may be mediated by the vestibular system (Cullen, 2012). The vestibulo-ocular reflex and musculoskeletal system control posture by integrating information from the vestibular cortex, visual cortex, and somatosensory system (Massion, 1998; van der Kooij et al., 1999). Vestibular deterioration and dysfunction are related to poor balance and cognitive decline (Smith and Zheng, 2013; Bigelow and Agrawal, 2015; D’Silva et al., 2016). Nonetheless, the mechanisms underlying this association and strategies to prevent cognitive impairment should be further studied.

This study has strengths. First, participants were from a large national prospective study conducted in China, and standardized protocols and rigid quality control procedures were adopted in the surveys. Second, a longitudinal methodology was used to assess whether standing balance predicted changes in cognition over a long follow-up period.

This study has limitations. First, although the regression models adjusted for demographics, health behaviors, and chronic diseases, other covariates, including genetic factors, were not considered in the analyses. Second, some participants were excluded because of missing data, potentially leading to selection bias. Third, the cognitive function test did not measure the level of cognitive impairment. Therefore, additional studies are needed to investigate the relationship between standing balance and the level of cognitive function.

## Conclusion

Poor balance was associated with lower cognitive function among middle-aged and older Chinese adults. Moreover, lower balance test scores were associated with a higher decline in episodic memory, mental status, and global cognition. The tandem test is simple and inexpensive and can assess the risk of cognitive decline. Nonetheless, more research is needed to identify the causes of cognitive and balance deficits. In addition, a better understanding of the link between postural balance and cognition can improve the diagnosis and management of these deficits.

## Data availability statement

Publicly available datasets were analyzed in this study. This data can be found here: http://opendata.pku.edu.cn.

## Ethics statement

Ethical review and approval was not required for this study in accordance with the local legislation and institutional requirements. The CHARLS data on which this study was based were reviewed and approved by Peking University’s Biomedical Ethics Review Committee (IRB00001052-11015). The patients/participants provided their written informed consent to participate in this study.

## Author contributions

JY, FL, and MW choose the research topic and designed the study. WD, XZ, and ML analyzed the data. JY performed the statistical analysis, assisted by FL and ML. JY and FL drafted the manuscript. YC, MW, WD, and XZ reviewed the manuscript. All authors approved the version to be published.
